# Molecular and genetic targets within metastatic colorectal cancer and associated novel treatment advancements

**DOI:** 10.3389/fonc.2023.1176950

**Published:** 2023-06-20

**Authors:** Christopher G. Cann, Michael B. LaPelusa, Sarah K. Cimino, Cathy Eng

**Affiliations:** ^1^ Department of Medicine: Hematology/Oncology, Vanderbilt University Medical Center, Nashville, TN, United States; ^2^ Department of Pharmacy, Vanderbilt University Medical Center, Nashville, TN, United States

**Keywords:** EGFR inhibitor, KRAS, metastatic colorectal cancer, HER2, mismatch repair

## Abstract

Colorectal cancer results in the deaths of hundreds of thousands of patients worldwide each year, with incidence expected to rise over the next two decades. In the metastatic setting, cytotoxic therapy options remain limited, which is reflected in the meager improvement of patient survival rates. Therefore, focus has turned to the identification of the mutational composition inherent to colorectal cancers and development of therapeutic targeted agents. Herein, we review the most up to date systemic treatment strategies for metastatic colorectal cancer based on the actionable molecular alterations and genetic profiles of colorectal malignancies.

## Introduction

Within the United States, colorectal cancer (CRC) continues to be a substantial source of morbidity and mortality, with an estimated 153,000 new cases diagnosed and over 52,000 deaths projected in 2023 alone (1). Nearly a quarter of patients are afflicted with metastatic disease (mCRC) at disease presentation, while another 20% of patient initially diagnosed with localized disease, progressing to stage IV disease (2, 3). Stage IV disease portends a very poor prognosis, with an estimated 5-year survival rate of only 14%. While survival rates have improved within the United States and globally over the past several decades for CRC of all stages, mCRC survival rates have remained stable without significant progress (3–5). Therefore, extensive comprehension of the varying molecular and genetic profiles within mCRC and development of associated anti-neoplastic targets is pivotal to treatment advancement and improving patient outcomes. We present a review of the most current, trial-based evidence of the treatment of mCRC based on unique molecular and genetic profiles that allow for refinement and strengthening of therapeutic options for patients limited cytotoxic therapy options.

## EGFR inhibitors

### The role of EGFR in cellular signaling and its inhibition

The propagation of many known human neoplasms are driven by activation of epidermal growth factor receptor (EGFR) and its subsequential signaling pathways (6). Binding of an activating ligand to EGFR results in phosphorylation of EGFR tyrosine kinase, triggering downstream signaling pathways involved in cellular proliferation and metabolism. EGFR is involved in several pathways, including the phosphatidylinositol 3-kinase (PI3K)/Akt/mammalian target of rapamycin (mTOR) pathway as well as the RAS/RAF/mitogen-activated protein kinase (MAPK) pathway (7). Activation or dysregulation of the of these pathways or imbalance of the sensitive feedback loops results in transcription of genes promoting cell survival, anti-apoptosis, proliferation, angiogenesis and metastatic potential (6, 8).

Cetuximab and panitumumab are monoclonal antibodies used in the treatment of metastatic colon cancer, directed against EGFR. Cetuximab is a chimeric IgG-1 monoclonal antibody while panitumumab is a recombinant humanized IgG-2 kappa monoclonal antibody, both working to competitively inhibit the extracellular ligand of EGFR, limiting the aforementioned abnormal cellular signaling that result in tumorigenesis (9). Although considered equivalent in their efficacy, cetuximab has been shown to have a higher incidence of hypersensitivity reactions, with an estimated risk ratio of 5.47. This hypersensitivity was shown likely to be secondary to previously developed IgE antibodies against galactose-alpha-1,3-galactose present on the Fab portion of the cetuximab heavy chain. The prevalence of this pre-existing IgE antibody is higher in the Southeastern United states, thought related to regional exposure (10).

### EGFR inhibitors and efficacy based on *RAS* mutational status

Mutations in genes (notably *KRAS*, *NRAS*, and *BRAF*) that encode proteins involved in EGFR-mediated cellular signaling pathways are associated with a lack of response to anti-EGFR therapy in mCRC (11–17). Mutations in the *RAS* family of genes result in protein expression that lead to inappropriate constitutive activation of the RAS/RAF/MAPK signaling that is less likely to be affected by inhibition of the upstream interaction of EGFR with an activating ligand. Thus, testing for these mutations is essential to ensure patients whose tumors harbor these mutations are not subjected to ineffective therapy with potentially severe toxicity and expense.

The first study to evaluate the use of EGFR inhibition in mCRC was in 2008, comparing the cetuximab use of cetuximab versus best supportive care (**Table 1**). The authors found that patients with wild type KRAS tumors had a significantly improved OS (9.5 *vs* 4.8 months HR0.55; 95% CI 0.41-0.74) with the use of cetuximab, versus no difference in survival or PFS for those with KRAS mutated tumors (**Table 1**). This was followed by studies investigating EGFR inhibition in combination with cytotoxic chemotherapy (14).

**Table 1:** Pivotal Clinical Trials in Metastatic Colorectal Cancer categorized by tumor characteristics.

Trial Name	Target Tumor Characteristics	Therapy Line	Arms	Primary Outcomes	Secondary Outcomes
Open-Label Phase III Trial (NCT00113763)	*KRAS*	Second and beyond	Investigational Arm: Panitumumab as an intravenous (IV) infusion at a dose of 6 mg/kg once every 2 weeks until participants develop progressive disease or are unable to tolerate study drug. Participants will also receive best supportive care as judged appropriate by the investigator and according to institutional guidelines Comparison Arm: Best supportive care	Median Progression Free Survival (mPFS): 8.0 v 7.3 weeks; Hazard Ratio (HR): 0.54 (95% CI, 0.44 to 0.66; p < 0.0001)	Overall Survival (OS): 30 vs 31 weeks (HR: 1.00; 95% CI, 0.82 to 1.22)
CAN-NCIC-CO17 (NCT00079066)	*KRAS*	Refractory or ineligible for fluoropyrmidine, irinotecan and oxaliplatin	Arm A: Patients receive an initial loading dose of cetuximab IV over 120 minutes on day 1. Patients continue to receive maintenance infusions of cetuximab IV over 60 minutes weekly. Patients also receive best supportive care, defined as measures designed to provide palliation of symptoms and improve quality of life as much as possible. Arm B: Patients receive best supportive care as in Arm A. In both arms, treatment continues in the absence of disease progression or unacceptable toxicity.	*KRAS wt* Median Overall Survival (mOS): 9.5 vs 4.8, HR: 0.55 (95% CI, 0.41 to 0.74; p<0.001); no significant difference in *mutated KRAS* tumors OS reported	*KRAS wt* mPFS: 3.7 vs 1.9, HR:0.40 (95% CI, 0.03 to 0.54; p<0.001); no significant difference in *mutated KRAS* tumors PFS reported
PRIME (NCT00364013)	*KRAS Wild-type (wt)*	First	Investigational Arm: panitumumab IV infusion at a dose of 6 mg/kg on Day 1 and FOLFOX chemotherapy regimen on Days 1 and 2 of each 14-day cycle until disease progression or unacceptable toxicity Comparison Arm: FOLFOX chemotherapy regimen on Days 1 and 2 of each 14-day cycle until disease progression or until unacceptable toxicity	mPFS: 9.6 v 8.0 months; HR: 0.80 (95% CI, 0.66 to 0.97; p = 0.02)	mOS: 23.9 v 19.7 months; HR: 0.83 (95% CI, 0.67 to 1.02; p = 0.072)
FIRE-3 (NCT00433927)	*KRAS wt*	First	Arm A: standard standard FOLFIRI regimen consisting of 5-fluorouracil (5-FU), leucovorin and irinotecan plus cetuximab as an IV infusion of 400 mg/m2 at inittal infusion then 250 mg/m2 on day 1 and 8 of each cycle Arm B: standard FOLFIRI regimen plus bevacizumab as an IV infusion at a dose of 5mg/kg on day 1	Objective Response Rate (ORR): 62.0% v 58.0%; HR 1.18 (95% CI, 0.85 to 1.64; p = 0.18)	mPFS: 10.0 v 10.3 months, HR 1.06 (95% CI, 0.88 to 1.26; p = 0.55); mOS: 28.7 vs 25.0 months, HR 0.77 (95% CI, 0.62 to 0.96; p = 0.017)
CALGB/SWOG 80405 (NCT00265850)	*KRAS wt*	First	Investigational Arm: Patients receive cetuximab 400mg/m^2 IV over 2 hours on the first day of treatment, then 250 mg/m^2 IV over 1 hour weekly thereafter. Patients also receive either FOLFOX or FOLFIRI every two weeks as described in the intervention section. One cycle is defined as 8 weeks of treatment. Comparison Arm: bevacizumab 5 mg/kg IV every two weeks and then receive either FOLFOX or FOLFIRI every two weeks as described in the intervention section. One cycle is defined as 8 weeks of treatment.	mOS: 30.0 vs 29.0 months; HR 0.88 (95% CI, 0.77 to 1.01; p = 0.08)	mPFS: 10.5 v 10.6 months; HR 0.95 (95% CI, 0.84 to 1.08; p = 0.45)
PARADIGM (NCT02394795)	*KRAS wt*	First	Investigational Arm: 6mg/kg FOLFOX plus panitumumab Comparison Arm: 5mg/kg FOLFOX plus bevacizumab	mOS: 36.2 v 31.3; HR 0.84 (95% ci, 0.72 to 0.98; P = 0.030); *KRAS wt* left-sided tumors only mOS: 37.9 v 34.3 months; HR 0.82 (95.798% CI, 0.68 to 0.99; p = 0.031)	
20050181 (NCT00339183)	*KRAS wt*	Second	Investigational Arm: panitumumab as an IV infusion at a dose of 6 mg/kg plus a standard FOLFIRI regimen consisting of 5-fluorouracil (5-FU), leucovorin and irinotecan. Treatment was administered in cycles every two weeks Comparison Arm: standard chemotherapy regimen (FOLFIRI) consisting of 5-FU, leucovorin and irinotecan. Treatment is administered in cycles every two weeks	mPFS: 6.7 v 4.9 months; HR: 0.82 (95% CI, 0.69 to 0.97; p = 0.023);	mOS: 14.5 v 12.5 months; HR 0.92 (95% CI, 0.78 to 1.10; p = 0.37)
KRYSTAL-1 (NCT03785249)	*KRAS G12C mutated*	Chemotherapy-refractory	Phase dose exploration and tolerability of Adagrasib; combination dosing with Pembrolizumab, Cetuximab, or Afatinib	ORR: 46% (95% CI, 28 to 66) v 19% (95% CI, 8 to 33) ; mPFS: 6.9 (95% CI, 5.4 to 9.1) v 5.6 months (95% CI, 4.1 to 8.3)	mDOR: 7.6 (95% CI, 5.7 to not yet reached) v 4.3 months (95% CI, 2.3 to 8.3)
CodeBreaK 100 (NCT03600883)	*KRAS G12C mutated*	Chemotherapy-refractory	Phase dose exploration and tolerability of Sotorasib	ORR: 9.7% (95% CI, 3.6 to 19.9)	
MOUNTAINEER (NCT03043313)	*HER2+, RAS wt*	Chemotherapy-refractory	Cohort A (non-randomized): tucatinib twice per day orally on Days 1-21 and trastuzumab IV on Day 1. Cycles repeat every 21 days. Cohort B (randomized): tucatinib twice per day orally on Days 1-21 and trastuzumab intravenously (into the vein; IV) on Day 1. Cycles repeat every 21 days. Cohort C (randomized): tucatinib twice per orally every day. Participants who do not respond to therapy may have the option to receive tucatinib and trastuzumab.	ORR: 38% (95% CI: 28, 49)	mDoR: 12.4 months; 95% CI, 8.5 to 20.5 ; mPFS: 8.2 month (95% CI, 4.2 to 10.3); mOS: 24.1 months (95% CI 20.3 to 36.8)
HERACLES (NCT03225937)	*HER2 +, RAS wt*	Refractory or ineligble for fluoropyrmidine, irinotecan, oxaliplatin, EGFR inhibtors	Arm A: lapatinib 1000 mg daily per os + trastuzumab 4 mg/kg IV load, followed by 2 mg/kg IV weekly. Arm B: pertuzumab 840 mg iv load, followed by 420 mg iv Q3weeks + trastuzumab-emtansine 3.6 mg/kg iv on day 1 of each subsequent 3 week cycle.	Arm A: ORR: 28%.; Arm B: ORR: 9.7% (95% CI: 0 to 28)	Arm A: mPFS: 4.7 months (95% CI, 3.7–6.1). mOS: 10.0 months (95% CI, 7.9–15.8); Arm B: mPFS: 4.1 months (95% CI: 3.6 to 5.9) Stable Disease (SD): 67.7% (95% CI: 50 to 85)
BEACON (NCT02928224)	*BRAF V600E mutated*	Second and beyond	Investigational Triplet Arm:Encorafenib, (orally once daily) plus binimetinib (orally twice daily) plus cetuximab (standard of care regimen) Investigational Doublet Arm: Encorafenib (orally once daily) plus cetuximab (standard of care regimen) Comparison Arm: Cetuximab plus either FOLFIRI or irinotecan	Arm A: mOS: 9.0 v 5.4 months; HR 0.52; 95% CI, 0.39 to 0.70; p< 0.001 ; Arm B: mOS: 8.4 v 5.4 months; HR 0.60; 95% CI, 0.45 to 0.79; P < 0.001	
CheckMate 142 (NCT02060188)	MSI-H/dMMR	First-line	Cohort A: Nivolumab Monotherapy Cohort B: Nivolumab + Ipilimumab Cohort C: Nivolumab + Ipilimumab Cohort D: Nivolumab + Ipilimumab + Cobimetinib Cohort E: Nivolumab + BMS-986016 Cohort F: Nivolumab + Daratumumab	ORR: 69% (95% CI, 53 to 82)	DCR: 84% (95% CI, 70.5 to 93.6)
KEYNOTE-177 (NCT02563002)	MSI-H/dMMR	First-line	Investigational Arm: pembrolizumab 200 mg IV on Day 1 of each 21-day cycle for up to 35 treatments (approximately 2 years). Participants that have stopped the initial course of pembrolizumab and have stable disease but progress after discontinuation can initiate a second course of pembrolizumab for up to 17 cycles (approximately 1 year additional). Comparative Arm: Participants receive 1 of 6 possible standard chemotherapy regimens. Participants with documented disease progression following chemotherapy can crossover to receive pembrolizumab for up to 35 cycles (approximately 2 years). Participants that have stopped pembrolizumab and have stable disease but progress after discontinuation can initiate a second course of pembrolizumab for up to 17 cycles (approximately 1 year additional).	mOS: not reached v 36.7 months; HR 0.74 (95% CI, 0.53 to 10.3; p = 0.036); mPFS: 16.5 vs 8.2 months; HR 0.59 (95% CI, 0.45 to 0.79)	
CORRECT (NCT01103323)	No specified criteria	Chemotherapy-refractory	Investigational Arm: Regorafenib 160 mg per oral once daily for 3 weeks on 1 week off of every 4 week cycle plus Best Supportive Care Comparison Arm: placebo tablets per oral once daily for 3 weeks on 1 week off of every 4 week cycle plus Best Supportive Care	mOS: 6.4 v 5.0 months; HR 0.77 (95% CI, 0.64 to 0.96; p = 0.0005)	
FRESCO-2 (NCT04322539)	No specified criteria	Chemotherapy-refractory	Investigational Arm: Fruquintinib plus best supportive care Comparison Arm: Best supportive care	mOS: 7.4 v 4.8 months; HR 0.66 (95% CI, 0.55 to 0.80; p < 0.001)	mPFS: 3.7 v 1.8 months; HR 0.32 (95% CI, 0.27 to 0.39; p = 0.002)

**Figure 1:**
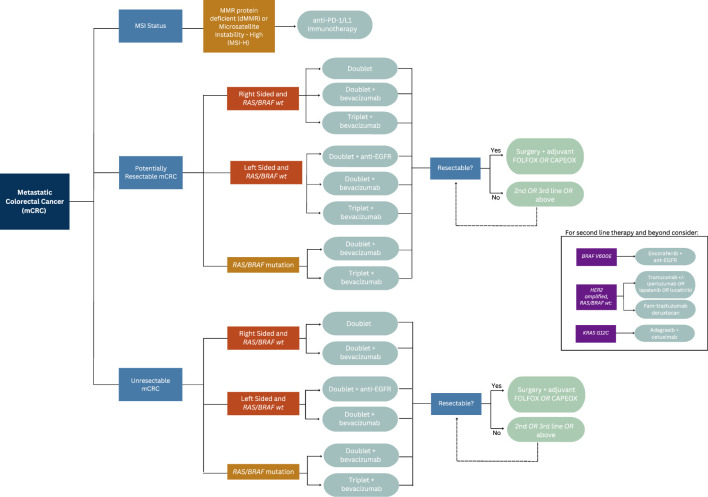
Flowchart depicting treatment options for mCRC based on tumor mutational status.

A retrospective analysis of three randomized controlled trials compared the outcomes of patients with mCRC who received chemotherapy or best supportive care with or without panitumumab in various lines of therapy, concluding patients with *KRAS* mutated mCRC were unlikely to benefit from EGFR inhibitors (**Table 1**) (18). Similarly, a subset analysis of patients enrolled in the CRYSTAL trial, which randomized untreated patients with mCRC to receive FOLFIRI either with or without cetuximab, found that patients with *KRAS* wild-type exon 2 tumors who received FOLFIRI and cetuximab experienced a longer median PFS compared to those who received FOLFIRI alone (9.9 *vs* 8.7 months; HR 0.68; 95% CI 0.50–0.94; p = 0.02) (12). An updated analysis of data from the CRYSTAL trial showed longer overall survival (OS) in patients who received cetuximab (23.5 *vs* 20.0 months; p = 0.009), a benefit largely derived by patients with *RAS* wild-type tumors (HR 0.69; 95% CI 0.54–0.88). Those with *RAS*-mutated tumors did not derive a survival benefit (1.05; 95% CI 0.86–1.28) (19, 20). This effect was also observed in the phase III PRIME trial, which compared groups of patients with untreated mCRC who received FOLFOX with or without panitumumab (**Table 1**). Among patients with wild-type *KRAS* and *NRAS* mCRC, improvements in PFS (HR 0.72; 95% CI 0.58–0.90; p = 0.004) and OS (HR 0.77; 95% CI 0.64–0.94; p = 0.009) were seen in patients who received FOLFOX plus panitumumab. Importantly, PFS was found to be worse in patients whose tumor harbored a *KRAS/NRAS* mutation (11, 21). The results reflect the current clinical practice of ensuring patients with *RAS* wild type tumors are provided anti-EGFR therapy, and that these therapies are avoided in those with a *RAS* mutated tumors due to lack efficacy or potentiation of worse outcomes. Please reference **Figure 1** for the suggested treatment algorithm based on mutational status.

### EGFR inhibition and primary tumor sidedness

The impact of EGFR inhibitors and the side of primary colon tumor is associated with treatment response, or lack thereof. This is believed to be a result of tumor sidedness being a surrogate for differing cumulative molecular subtypes (22).

In a multicenter analysis of 75 patients with *RAS* and *BRAF* wild-type mCRC who received cetuximab alone, panitumumab alone, or irinotecan plus cetuximab (in any line of therapy), no responses were observed in patients who had right-sided primary tumors while a response rate of 41% was seen in patients with left-sided primary tumors (p = 0.003). Progression free survival (PFS) (2.3 *vs* 6.6 months) was also longer in patients with left-sided tumors (HR 3.97; 95% CI 2.09–7.53; p < 0.0001) (23).

This phenomenon was demonstrated in retrospective evaluations of landmark CRC trials. First, in the CRYSTAL trial, referenced above, which randomized patients to FOLFIRI plus cetuximab versus FOLFIRI alone, and the FIRE-3 trial comparing FOLFIRI plus cetuximab versus FOLFIRI plus bevacizumab, patients with *RAS* wild type left sided tumors had had superior objective response rate (ORR), PFS and OS with the addition of cetuximab, in contrast to minimal efficacy seen in right sided wild type tumors (**Table 1**) (12, 24, 25). The CALGB/SWOG 80405 not only demonstrated poorer prognosis associated with right sided CRC, but *KRAS* wild type right sided tumors had significantly worse median OS relative to left sided *KRAS* wild type tumors when treated with cetuximab and chemotherapy (16.7 months (95% CI 13.1-19.4) *vs* 36 months (95% CI 32.6-40.3) (**Table 1**) (26). Most recently, the phase III, open-label multicenter PARADIGM trial was designed to determine the superiority of anti-EGFR therapy or anti-vascular endothelial growth factor (anti-VEGF) therapy when added to modified FOLFOX6 in *RAS* wild type mCRC (**Table 1**). This showed an improvement in overall survival by 3.6 months in patients with left sided tumors that were treated with panitumumab compared to bevacizumab (27).

### EGFR inhibitors and conversion to resectable disease

EGFR inhibitors, when combined with chemotherapy, have also shown an ability to increase the possibility of liver metastases resection in patients with *RAS* wild-type mCRC. In a randomized trial from China, the addition of cetuximab to chemotherapy resulted in 20 of 70 (29%) of patients becoming eligible for hepatic resection compared to 9 of 58 (13%) of patients who did not receive cetuximab. R0 resection rates were 25.7% in cetuximab arm compared to 7.4% in those who didn’t receive cetuximab (p <0.01). Additionally, surgery improved median survival compared to those who did not receive surgery in the cetuximab arm (46.6 *vs* 25.7 months; p = 0.007) and the control arm (36.0 *vs* 19.6 months; p = 0.016) (28). In the VOLFI phase II trial, 75% of patients with *RAS* wild-type mCRC and liver metastases deemed potentially resectable were successfully converted to resectable disease upon receiving FOLFOXIRI with panitumumab compared to 36.4% in group of patients who received FOLFOXIRI alone. ORR was higher in the panitumumab arm, while PFS and OS were similar between both arms, with OS trend in favor of the arm that received panitumumab (29).

### EGFR inhibitors in patients with refractory disease

For patients with wild-type *KRAS/NRAS/BRAF* mCRC whose disease progressed on a therapeutic regimen that contained an EGFR inhibitor, the use of an EGFR inhibitor as part of therapy in the next line is generally not recommended. However, if these patients’ first-line regimen did not include an EGFR inhibitor, there is evidence that use of an EGFR inhibitor in the subsequent line of therapy is beneficial. For example, in a phase III trial analyzing wild-type *KRAS* exon 2 tumors that exhibited disease progression on oxaliplatin-based and irinotecan-based chemotherapy, panitumumab monotherapy was compared to best supportive care. This trial demonstrated an overall survival benefit of nearly 3 months with the use of panitumumab (10.0 *vs* 7.4 months; HR 0.73; 95% CI 0.57–0.93; p < 0.01) (30). Not all studies evaluating EGFR inhibitors in this setting have improved overall survival, however. In Study 20050181, the addition of panitumumab to FOLFIRI compared to FOLFIRI alone in patients with mCRC who had wild-type *KRAS* exon 2 tumors resulted in an improvement in median PFS (6.7 *vs* 4.9 months; HR 0.82; 95% CI 0.69–1.10; p = 0.023) but no difference in OS (median 14.5 *vs*. 12.5 months; HR 0.92; 95% CI 0.78–1.10; p = 0.37) (**Table 1**) (31, 32). In the EPIC trial, which compared irinotecan plus cetuximab to irinotecan alone as second line treatment in patients with mCRC who progressed on first-line fluoropyrimidine and oxaliplatin based therapy, both ORR and median PFS, were significantly improved in the combination group (PFS 5.4 *vs* 2.6 months (95% CI 0.46-0.69), ORR 29.4% *vs* 5.0% (95% CI 4.04-17.40) respectively). There was no statically significant difference in median OS between arms, however, a post study treatment analysis indicated improvement in OS in those who received post-study cetuximab relative to those who received subsequent therapy without cetuximab or no therapy at all. Importantly, quality of life was found to be improved in the combination arm, including improvement in physical functioning, nausea, vomiting, appetite loss and pain (33, 34).

### Chemotherapy choice when used in conjunction with EGFR inhibitors

There is conflicting data to suggest that oxaliplatin-based chemotherapy regimens reduce the efficacy of cetuximab in patients with untreated *RAS* wild-type mCRC. In the phase II OPUS trial, patients with mCRC and *KRAS* wild-type exon 2 tumors who received FOLFOX plus cetuximab in the first-line setting did not derive a statistically significant benefit with regard to OS compared to patients who received FOLFOX alone (22.8 *vs* 18.5 months; HR 0.85; p = 0.39) (16, 35). The lack of survival benefit when cetuximab is added to oxaliplatin-based regimens was also observed in the phase III MRC COIN trial, in which patients with *KRAS* wild-type mCRC or locally advanced disease who received cetuximab and either FOLFOX or CAPEOX did not have longer OS relative to patients who received chemotherapy alone (17.9 *vs* 17.0 months; HR 1.04; 95% CI 0.87–1.23; p = 0.67). However, a subgroup analysis of this trial indicated that those who received FOLFOX, rather than CAPEOX, might have experienced a benefit (36).

In contrast to the above findings, the results found by the phase III TAILOR trial, showed prolonged PFS (9.2 *vs* 7.4 months; p = 0.004) and OS (20.7 *vs* 17.8 months p = 0.02), and ORR (61.1% *vs* 39.5%; p < 0.001) among patients with untreated *RAS* wild-type mCRC who received cetuximab with FOLFOX compared to those who received FOLFOX alone (37). The results of the PARADIGM trial discussed above also demonstrated opposing results, with significantly improved OS in *RAS* wild type, left sided tumors with the use of oxaliplatin based therapy with the addition of panitumumab. Given these mixed results, suggested clinical practice is such that in the treatment of left sided *RAS* wild type tumors, the use of anti-EGFR therapy in conjunction with irinotecan or oxaliplatin based chemotherapy backbone is standard, with backbone choice based on individual patient co-morbidities and side effect profile.

### Efficacy of EGFR inhibition in patients with *BRAF* mutations

*BRAF* encodes a protein that functions downstream of *RAS* in the EGFR-mediated signaling pathway and, when mutated, is constitutively active (3, 27). Therefore, upstream EGFR inhibition alone is not thought to prevent abnormal signaling mediated by *BRAF* mutations.

Specifically, *BRAF V600E* mutations result in the inappropriate activation of MAPK independently of RAS (38). Abnormal regulation of these pathways is invariably linked to carcinogenesis (4). Given this downstream effect, inhibition of EGFR presents little utility in the setting of concurrent RAS wild type and BRAF mutated CRC.

Approximately five to nine percent of patients with mCRC have *BRAF* V600E mutations, which do not typically occur in co-existence with *RAS* mutations (39). In subset analyses of patients in the aforementioned PRIME trial, as well as the COIN trial, *BRAF* mutations were not found to be predictive of response to the combination of chemotherapy and an EGFR inhibitor in patients with untreated mCRC (24). One meta-analysis that included 463 patients with *BRAF* mutant mCRC including nine phase III trials and one phase II trial with mCRC concluded that the addition of an EGFR inhibitor did not improve PFS (HR 0.88; 95% CI 0.67–1.14; p = 0.33) or OS (HR 0.91; 95% CI 0.62–1.34; p = 0.63) (29). Another meta-analysis of seven randomized control trials (RCT) found EGFR inhibitors did not improve PFS (HR 0.86; 95% CI 0.61–1.21) or OS (HR 0.97; 95% CI 0.67–1.41) in patients with *BRAF* mutations (30). Therefore, the use of ant-EGFR therapy in the setting of BRAF mutations is of little to no efficacy.

## VEGF inhibitors

### The role of VEGF in cellular signaling

Vascular endothelial growth factor (VEGF) is a protein that, upon binding to VEGF receptors 1 and 2 on the surface of endothelial cells, promotes tumor angiogenesis by promoting permeability, survival, and proliferation of endothelial cells. VEGF is expressed in the majority of human malignancies, while having little role in normal physiological angiogenesis. The activity of VEGF is inhibited by bevacizumab, a humanized monoclonal antibody against circulating VEGF-A, that has become a mainstay adjunctive therapy in the treatment of mCRC (40, 41).

### Bevacizumab as part of first-line therapy

Several trials have investigated the efficacy of adding bevacizumab to chemotherapy in patients with untreated mCRC and have displayed varying results. Pooled results from several phase II trials of patients with untreated mCRC have indicated that OS was prolonged by the addition of bevacizumab to 5-flurouracil (5-FU)/leucovorin with or without irinotecan (42–44). A combined analysis of the results of these trials showed that adding bevacizumab to 5-FU/leucovorin improved median survival compared to 5-FU/leucovorin or irinotecan without bevacizumab (17.9 *vs* 14.6 months; p = 0.008) (45). In patients 70 years and older with untreated mCRC, the addition of bevacizumab to capecitabine prolonged PFS compared to capecitabine alone (9.1 *vs* 5.1 months; HR 0.53; 95% CI 0.41–0.69; p < 0.0001) in the AVEX trial (46).

### Chemotherapy choice when used in conjunction with bevacizumab

A meta-analysis of six RCTs encompassing a total of 3,060 patients showed that the addition of bevacizumab to chemotherapy in the first line setting prolonged PFS (HR 0.72; 95% CI 0.66–0.78; p < 0.00001) and OS (HR 0.84; 95% CI 0.77–0.91; p < 0.00001) relative to chemotherapy alone (47). Subgroup analyses, however, indicated that this addition was largely limited to patients who received irinotecan-based regimens. This result was also reflected in a SEER analysis which showed the addition of bevacizumab to oxaliplatin-based chemotherapy did not improve OS but did improve OS for patients who received irinotecan (48). Additionally, in a large phase III trial, PFS, but not OS, was prolonged by 1.4 months by addition of bevacizumab to oxaliplatin-based chemotherapy (HR 0.83; 97.5% CI 0.72–0.95; p = 0.0023) in patients with untreated mCRC, yet a subset analysis suggested that those who received CAPEOX (rather than FOLFOX) were most likely to experience that benefit (49). To date, no trials have compared FOLFIRI to FOLFIRI plus bevacizumab or FOLFOXIRI to FOLFOXIRI plus bevacizumab. Despite the results discussed above, clinical practice prioritizes the use of bevacizumab in those with RAS mutant or right sided RAS wild type metastatic colon cancers in patients without contraindications to its use.

### Bevacizumab and conversion to resectable disease

Few trials have been conducted to investigate the utility of bevacizumab in the peri-operative setting. The BECOME trial specifically evaluated the role of bevacizumab, in conjunction with FOLFOX, in the conversion of unresectable mCRC to resectable disease in patients with unresectable liver-limited mCRC. This trial found that the addition of bevacizumab to FOLFOX improved the rate at which patients underwent R0 hepatic resection (22.3% *vs* 5.8%; p < 0.01) (50). The multinational phase II OLIVIA trial sought to evaluate if the role of bevacizumab to either FOLFOX or FOLFOXIRI to facilitate oligometastatic resection in patients initially determined to have unresectable liver metastasis. The combination of FOLFOXIRI with bevacizumab resulted in higher ORR (81% (95% CI 65-91) *vs* 62% (95% CI 45-77), rate of resection (61% (95% CI 45-76) *vs* 49% (95% CI 32-65)), R0 resection rate (49% *vs* 23%) and median PFS (18.6 (95% CI 12.9-22.3) *vs* 11.5 months (95% CI 9.6-13.6)) relative to bevacizumab plus FOLFOX. These response rates were at the expense of higher grade ≥3 adverse events, including neutropenia (50% *vs* 35%), febrile neutropenia (13% *vs* 8%), and diarrhea (30% *vs* 14%) (51).

In the post-operative setting, the HEPATICA trial was designed to evaluate DFS in patents with mCRC who received CAPEOX with or without bevacizumab after resection of liver metastases. Unfortunately, due to low accrual and subsequent study closure no statistically significant conclusion was able to be drawn. However, the group who received CAPEOX and bevacizumab demonstrated higher scores related to quality of life than patients who received CAPEOX alone (52).

### Bevacizumab as maintenance therapy

The utility of administering bevacizumab after disease stability has been achieved with chemotherapy-based regimens has been studied in several large trials with conflicting results. The CAIRO3 trial, analyzing patients with mCRC deemed to have at least stable disease after first-line treatment with CAPEOX and bevacizumab, were assigned to receive either maintenance capecitabine plus bevacizumab or observation. At time of progression, patients in both groups subsequently received CAPEOX plus bevacizumab until their disease progressed further. The study found that time to second progression was improved in patients who received maintenance capecitabine plus bevacizumab compared to those who were randomized to observation (8.5 *vs* 11.7 months; HR 0.67; 95% CI 0.56–0.81; p < 0.0001). No significant difference in OS was observed, although a trend towards improved OS was seen in patients who received maintenance capecitabine plus bevacizumab (53, 54). AIO 0207 trial showed bevacizumab alone was non-inferior to fluorouracil plus bevacizumab in time to first progression (HR 1.08; 95% CI 0.85–1.37; p = 0.53). Additionally, this study indicated that no treatment in the maintenance setting was not non-inferior to either bevacizumab alone or fluorouracil plus bevacizumab in patients who previously received induction therapy with oxaliplatin-based chemotherapy plus bevacizumab (55).

The previously mentioned data supporting maintenance bevacizumab conflicts with the outcome of PRODIGE9, which found that bevacizumab did not improve tumor control duration (15.08 *vs* 14.98 months HR 1.09; 95% CI 0.87–1.37), PFS (9.20 *vs* 8.90 months; HR 0.92; 95% CI 0.76–1.10), or OS (21.65 *vs* 21.98 months; HR 1.05; 95% CI 0.86–1.28) relative to no maintenance treatment among patients initially treated with FOLFIRI and bevacizumab (56). Similarly, the SAKK 41/06 trial found that non-inferiority in time to progression was not reached when comparing maintenance bevacizumab to no maintenance treatment in patient previously receiving previous chemotherapy plus bevacizumab (4.1 *vs* 2.9 months; HR 0.74; 95% CI 0.58–0.96) (57).

Maintenance bevacizumab was compared to maintenance bevacizumab plus erlotinib, an EGFR inhibitor, in the GERCOR DREAM; OPTIMOX3 trial. Median PFS from maintenance was not significantly different but trended towards use of both drugs (5.4 *vs* 4.9 months; stratified HR 0.81; 95% CI 0.66–1.01; p = 0.059) while median OS from maintenance was longer in patients that received both bevacizumab and erlotinib (24.9 *vs* 22.1 months; stratified HR 0.79; 95% CI 0.63–0.99; p = 0.036). However, Grade 3-4 adverse effects occurred in 21% of patients who received bevacizumab plus erlotinib compared to 0% of patients who received bevacizumab alone (58). Due to these significantly higher adverse effects of this combination in the setting of non-curative disease, the erlotinib is not routinely used in the maintenance setting. In clinical practice, largely based on the CAIRO3 study, de-escalated chemotherapy plus bevacizumab is safely and effectively used in the maintenance setting.

### Bevacizumab in patients with refractory disease

Single agent bevacizumab is not recommended after progression on chemotherapy is generally not recommended due to inferior efficacy compared to chemotherapy alone or chemotherapy plus bevacizumab. Several trials have evaluated the efficacy of bevacizumab, in conjunction with chemotherapy, in patients with mCRC who experienced progression on first-line chemotherapy. In the ML18147 trial, patients with mCRC who progressed on first-line chemotherapy and bevacizumab were subsequently randomized to a different chemotherapy backbone with or without bevacizumab. Patients who were provided bevacizumab saw a statistically significant OS benefit (11.2 *vs* 9.8 months; HR 0.81; 95% CI 0.69–0.94; p = 0.0062) (59). The benefit of continuing bevacizumab, with a different chemotherapeutic regimen, in the second-line setting after progression on a regimen containing bevacizumab was also observed in the BEBYP trial, noting a longer PFS in patients who were continued on a regimen that contained bevacizumab (6.8 *vs* 5.0 months; HR 0.70; 95% CI 0.52–0.95; p = 0.001) (60). Further, adding bevacizumab to second-line FOLFOX for patients with mCRC who progressed on first-line irinotecan-based therapy that did not include bevacizumab was the focus of Study E3200. An improvement in median duration of survival was seen in the patients treated with second line FOLFOX plus bevacizumab compared to FOLFOX alone (12.9 *vs* 10.8 months; HR 0.75; p = 0.0011) (61). Retrospective and observational analyses also concur that continuation of bevacizumab after progression first-line chemotherapy containing bevacizumab provides a survival benefit (62, 63).

### Ziv-aflibercept

Ziv-aflibercept is a recombinant protein designed to inhibit angiogenesis by preventing VEGF -A, B and placental growth factor from activating VEGF receptors. This novel drug evaluated in the phase III VELOUR trial studying its use in conjunction with FOLFIRI in patients with mCRC who had prior disease progression on oxaliplatin-based chemotherapy. OS was longer in patients who received FOLFIRI and ziv-aflibercept compared to FOLFIRI alone (13.5 *vs* 12.1 months; HR 0.82; 95% CI 0.71–0.94; p = 0.003) (64). Overall, clinical practice favors bevacizumab use in this setting due to its superior toxicity profile and lower cost.

### Ramucirumab

Ramucirumab, a human IgG-1 monoclonal antibody against the extracellular portion of the VEGF receptor 2, has been studied in the chemotherapy refractory setting combined with cytotoxic regimens. In the phase III RAISE trial, patients with mCRC who had disease progression on FOLFOX and bevacizumab were randomized to FOLFIRI with or without ramucirumab. Patients in the ramucirumab arm experienced longer OS (13.3 *vs* 11.7 months; HR 0.84; 95% CI 0.73–0.98; p = 0.02) although therapy was discontinued more frequently in the group that received ramucirumab (11.5% *vs* 4.5%), most frequently secondary to neutropenia, thrombocytopenia, stomatitis and diarrhea (65). As a result of this study, the addition of ramucirumab to irinotecan or FOLFIRI for patients with refractory mCRC not previously exposed to irinotecan-based therapy is considered an acceptable regimen. However, bevacizumab remains most utilized clinically.

### Regorafenib

Regorafenib is a multi-targeted tyrosine kinase inhibitor (TKI) that blocks interactions of ligands with VEGF, PDGF, BRAF, KIT, and RET and has been studied primarily in patients with refractory mCRC. Its broad receptor influence modulates downstream pathways involved in angiogenesis, cell growth, differentiation, and survival. The CORRECT trial evaluated the administration of regorafenib or placebo to patients with refractory mCRC whose disease had progressed on several lines of chemotherapy (**Table 1**). The study indicated prolonged OS in patients who received regorafenib (6.4 *vs* 5.0 months; HR 0.77; 95% CI 0.64–0.94); p = 0.005) (66). The CONCUR trial conducted in Asia observed this similar outcome, with prolonged OS with use of regorafenib compared to placebo in the refractory setting (8.8 *vs* 6.3 months; HR 0.55; 95% CI 0.40–0.77; p < 0.001) (67). Hand-foot skin reaction was the most frequent grade 3 (or higher) adverse effect and occurred in 17% of patients who received regorafenib in this trial. Other, but less common grade 3 (or higher) adverse effects included fatigue, hypertension, diarrhea, rash/desquamation. The ReDos trial utilized a dose-escalation of regorafenib to mitigate toxicity, while maintaining efficacy, however, adverse events remained significant (68). Due to the findings in these two trials, regorafenib is considered an accepted treatment regimen for patients with mCRC whose disease has progressed on chemotherapy, but its side effect profile warrants careful monitoring while on therapy.

### Fruquintinib

Fruquintinib is a highly selective TKI that blocks VEGFR-1, VEGFR-2, and VEGFR-3 which was recently evaluated in the phase III FRESCO 2 trial, which randomized patients with refractory, previously treated mCRC (**Table 1**). Patients were allowed to have received prior trifluridine/tiparicil and/or regorafenib (median lines of therapy 5) to receive either best supportive care with or without fruquintinib. Patients who received fruquintinib experienced prolonged OS (7.4 *vs* 4.8 months; HR 0.66; 95% CI 0.55–0.80; p < 0.001 and PFS (3.7 *vs* 1.8 months; HR 0.32; 95% CI 0.27–0.39; p < 0.001). Grade 3 or higher adverse effects were seen in 62.7% of patients who received fruquintinib compared to 50.4% in patients who received placebo. Specific side effects seen in over 5% of patients were hand-foot syndrome, asthenia, and hypertension (69). Importantly, 97% of enrolled patients had received prior bevacizimab. Fruquitinib can be used after progression on other VEGF inhibitors including bevacizumab and regorafenib.

### EGFR inhibitors versus bevacizumab

*RAS* mutational status and tumor sidedness impact the efficacy of bevacizumab and EGFR inhibitors in the first-line setting. As previously mentioned, in the CALGB/SWOG 80405 trial, no statistically significant OS benefit (30.0 *vs* 29.0 months; HR 0.88; 95% CI 0.77–1.01; p = 0.08) was seen among patients with wild-type *KRAS* exon 2 mCRC who received first-line chemotherapy (either FOLFOX or FOLFIRI) with cetuximab versus bevacizumab (70). However, patients with *RAS* wild-type, right-sided mCRC who received bevacizumab in the first-line setting showed a trend toward longer OS than those who received cetuximab (HR 1.36; 95% CI 0.93–1.99; p = 0.10). Conversely, patients with *RAS* wild-type, left-sided primary tumors who received cetuximab had significantly longer overall survival than those who received bevacizumab (HR 0.77; 95% CI 0.59–0.99; p = 0.04) (71).

In contrast, the FIRE-3 trial found an improvement in OS among patients who received first line FOLFIRI plus cetuximab compared to FOLFIRI plus bevacizumab (28.7 *vs* 25.0 months; HR 0.77; 95% CI 0.62–0.96; p = 0.017) in patients with *KRAS* exon 2 wild type mCRC (24, 72). However, trial has been criticized for its lack of third-party review and low rate of administration of second-line therapy (70). Improved efficacy with an EGFR inhibitor was also seen in the phase II PEAK trial, in which patients with wild-type *RAS* who received FOLFOX with panitumumab had longer PFS (12.8 *vs* 10.1 months; HR 0.68; 95% CI 0.48–0.96; p = 0.029) than patients who received FOLFOX and bevacizumab, although some have argued the small sample size limit its generalizability (73, 74). The more recent PARADIGM trial, discussed above, which compared FOLFOX plus panitumumab to FOLFOX plus bevacizumab in the first line for patients with *RAS* wild-type mCRC, showed longer OS for patients with left sided tumors using panitumumab (37.9 *vs* 34.3 months; HR 0.82; 95% CI 0.68–0.99; p =. 0.031) (27).

In the second-line setting, there is a paucity of data comparing bevacizumab and EGFR inhibitors. In the phase II SPIRITT trial, treatment with FOLFIRI plus panitumumab did not yield longer PFS survival compared to FOLFIRI plus bevacizumab in patients with *KRAS* wild type mCRC whose disease progressed on first-line oxaliplatin-based chemotherapy and bevacizumab (7.7 months *vs* 9.2 months; HR 1.01; 95% CI 0.68–1.50; p = 0.97) (75).

### Combination EGFR and VEGF inhibition

The combination of EGFR and VEGF inhibition has shown efficacy in preclinical setting, finding improved survival and tumor inhibition in mouse models (76, 77). Given these findings and the proven benefit of the addition of EGFR or VEGF to cytotoxic therapy, investigators sought to determine the utility of VEGF in conjunction EGFR therapies in the metastatic setting.

The addition of bevacizumab and panitumumab to chemotherapy in first-line treatment of patients with mCRC (of all *KRAS* mutational subtypes) was studied in the phase III PACCE trial. Patients received chemotherapy and bevacizumab with or without panitumumab. The addition of panitumumab resulted in higher toxicity and shorter PFS (10.0 *vs* 11.4 months; HR 1.27; 95% CI 1.06–1.52), regardless of *KRAS* mutational status (78). The CAIRO2 trial came to a similar conclusion, with the addition of cetuximab to CAPEOX plus bevacizumab yielded a higher incidence of grade 3-4 toxicity (81% *vs* 72%; p = 0.03) and shorter PFS (9.4 *vs* 10.7 months; HR 1.22; 95% CI 1.04–1.43) (79). No difference in PFS between groups was observed among patients with wild-type KRAS tumors.

Conversely, the phase II randomized BOND-2 study investigated the use of cetuximab and bevacizumab in irinotecan-refractory mCRC. This study indicated that the addition of cetuximab and bevacizumab to irinotecan in this patient population resulted in improved time to progression (7.3 *vs* 4.9 months), improved response rate (37% *vs* 20%) and an overall survival benefit (14.5 *vs* 11.4 months) relative to cetuximab and bevacizumab alone, and without unexpected or higher rates of toxicity (80).

Due to the incidence of adverse effects experienced by patients in the PACCE and CAIRO2 trials, as well as the lack of efficacy, it is not recommended to combine these two drug classes within the same line of therapy.

## BRAF inhibitors

### Treatment for *BRAF* V600E mutation positive disease in non-first line setting

Inhibition of BRAF has been primarily studied in second line or greater setting. For patients with mCRC whose tumors contain *BRAF* V600E mutations with progression on first or second-line therapy, a triplet of therapy comprising encorafenib, a BRAF inhibitor, plus binimetinib, a MEK inhibitor, and cetuximab was compared to the doublet of encorafenib and cetuximab as well as to cetuximab plus either irinotecan or FOLFIRI in the BEACON trial (**Table 1**). Treatment with the triplet or doublet led to an OS benefit relative to treatment with cetuximab plus either irinotecan or FOLFIRI (9.3 *vs* 9.3 *vs* 5.9 months, respectively). Grade 3 adverse effects occurred more commonly in patients who received the triplet than those who received the doublet (58% *vs* 50%). Therefore, to limit toxicity while maintaining efficacy, doublet therapy (encorafenib plus either cetuximab or panitumumab) is recommended (81).

Irinotecan plus cetuximab and vemurafenib, a BRAF inhibitor, was evaluated in the treatment refractory setting, indicating improvement in PFS and disease control rate compared to irinotecan plus cetuximab alone in this population (82). To mitigate EGFR-mediated adaptive feedback reactivation of MAPK signaling, different combinations of dabrafenib, a BRAF inhibitor, panitumumab, and trametinib, a MEK inhibitor, were studied in patients with *BRAF* V600E mutation positive mCRC, with variable response rates. The triplet combination of these therapies was found to have the highest response rate (21%), but has not been adopted as a standard of care (83).

### BRAF inhibitors in the first-line setting

Due to the significantly worse OS and limited response to standard first line therapy of BRAF mutated mCRC, BRAF inhibitors are also being studied in the first-line systemic therapy for patients with *BRAF* V600E mutated mCRC. The BREAKWATER trial (NCT040607421) is a phase 3 trial investigating the efficacy and safety of encorafenib, cetuximab, and either FOLFIRI or FOLFOX in patients with untreated *BRAF* V600E mutated mCRC. Additionally, the SEAMARK trial (NCT05217446) is a phase 2 trial comparing the combination of encorafenib, cetuximab, and pembrolizumab, an inhibitor of programmed death-1 receptor, to pembrolizumab alone in patients with untreated deficient mismatch repair (dMMR) and *BRAF* V600E mutated mCRC. Results are still pending for both trials.

## Anti-HER2 therapy

### HER2 in colorectal cancer

Human epidermal growth factor receptor 2 (HER2), which is encoded by the proto-oncogene *ErbB2* (also known as *HER2*), is a member of the same family of signaling kinase receptors as EGFR. Dimerization of HER2 with other members of the EGFR family results in activation of several downstream signaling pathways, including RAS/RAF/ERK, PI3K/AKT/mTOR, and JAK/STAT3 (84, 85). *HER2* is not commonly amplified or overexpressed in CRC with a prevalence estimated at 3 to 5%, however, is more frequently amplified or overexpressed in *RAS*/*BRAF* wild type tumors (86). HER2 has become one of the latest areas of study in targeted medicine within colorectal cancer. HER2 amplification or overexpression may predispose to the development of resistance upon treatment with an EGFR inhibitor for patients with *RAS*/*BRAF* wild type mCRC (83, 87). The prognostic value of HER2 expression or amplification is not well defined, however attempts to understand its impact have been performed. Specifically, In a cohort of patients with *RAS*/*BRAF* wild type mCRC whose treatment regimen included an EGFR inhibitor, median PFS was shorter among those with HER2 amplification compared to those without HER2 amplification (2.8 *vs* 8.1 months; HR 7.05; 95% CI 3.4–14.9; p < 0.001) (88). At this time, HER2-directed therapy is generally recommended in patients with *HER2*-amplified mCRC whose disease has progressed on systemic cytotoxic therapy, only to be considered first-line for patients who are not appropriate for cytotoxic therapy.

### Trastuzumab-based therapy

The combination of two HER2-directed monoclonal antibodies, trastuzumab and pertuzumab, has been studied in two basket studies of patients with HER2-amplified cancers. In refractory HER2-amplified mCRC, an ORR of 23.1% (95% CI 18.1%–28.7%) and DCR of 44.2% (95% CI 38.1%–50.5%) was observed among 57 patients in the MyPathway study while an ORR of 14% (90% CI 4%–33%) and disease control rate of 50% (90% CI 36%–60%) was seen in 28 patients in the TAPUR study. Grade 3 or 4 AEs were limited, noted in up to 37% of patients in the MyPathway study while two patients in the TAPUR study developed grade 3 AEs (86, 89).

Trastuzumab has also been studied in combination with several other agents in this setting. The phase II HERACLES trial studied 27 patients with refractory HER2-positive, KRAS wild type mCRC who received trastuzumab plus the oral tyrosine kinase inhibitor lapatinib targeting EGFR1 and HER2 (**Table 1**). Nearly one third of patients had an object response (30% 95% CI 14%–50%), with 22% of patients experiencing grade 3 AEs, without any grade 4 events (90–92). Additionally, the efficacy of fam-trastuzumab deruxtecan, an antibody drug conjugate containing anti-HER2 antibody and a cytotoxic topoisomerase I inhibitor linked by a cleavable tetrapeptide linker, was the focus of the phase II DESTINY-CRC01 trial. 78 patients with refractory HER2-expressing, *BRAFV600E* and *RAS* wild type mCRC were stratified into three groups based on HER2 expression. Responses were only seen in patients with high tumoral HER2 expression (IHC3+ or IHC2+/ISH+), with an ORR of 45.3% (95% CI 31.6%–59.6%) and PFS 6.9 months (95% CI 4.1–8.7 months). Importantly, these responses were seen regardless of previous exposure to HER2 directed therapy. Unfortunately, 65.1% of the studied patients experienced grade 3 or higher AEs. Specifically, 9% of patients developed life threatening interstitial lung disease, with 3 fatalities (93).

More recently, The MOUNTAINEER trial evaluated the combination of trastuzumab and the HER2 selective tyrosine kinase inhibitor tucatinib (**Table 1**). Over 100 patients with refractory HER2-positive, *RAS* wild type mCRC were stratified to receive trastuzumab plus tucatinib or tucatinib monotherapy, with cross over permitted to the combination arm upon progression. 84 patients received trastuzumab and tucatinib, with an ORR of 38.1%, median duration of response of 12.4 months, median PFS of 8.2 months, and median OS of 24.1 months. Tucatinib monotherapy had a limited objective response of 3%, with no PFS or OS reported due to extensive cross over into the combination arm. This regimen had a superior side effect profile relative to other HER2 directed strategies, noting minimal grade 3 events, only 5 patients discontinuing therapy due to adverse effects, and no treatment related deaths (94). The results led to expedited FDA approval for this combination in refractory mCRC, and the phase III MOUNTAINEER-03 trial (NCT05253651), is ongoing, comparing trastuzumab plus FOLFOX to either FOLFOX, FOLFOX plus bevacizumab, or FOLFOX plus cetuximab for patients with untreated HER2-positive mCRC.

## KRAS G12C

With the recognition of inferior outcomes utilizing EGFR inhibition in *KRAS* mutated CRC, it has become standard of care to test for RAS mutations *via* next generation sequencing prior to initiation of systemic therapy if possible. It is estimated that half of CRC harbor a KRAS mutation, varying in frequency amongst ethnicities KRAS mutation, with multiple studies suggesting associated worse prognosis (95–99).

A specific mutation within this family, *KRASG12C*, found in an estimated 3% of metastatic CRC, has shown to have poorer OS relative to other KRAS mutated CRC by up 10 months (99). However, this mutation has recently been found to be a valuable target for systemic therapy across various histologies and within CRC. CodeBreaK100, a phase II single arm trial published in 2021, used the irreversible *KRASG12C* protein inhibitor sotorasib in solid tumors harboring the KRASG12C mutation, including 62 CRC patients previously treated with 5-FU, oxaliplatin and irinotecan. In the CRC cohort, a modest 9.7% of patients had an objective response, not reaching primary endpoint of an 20% objective response rate (100).

This lack of response in the CRC relative to other histologies such as non-small lung cancer, is related to several factors including upstream basal receptor tyrosine kinase activation interfering with *KRASG12C* inhibitors and feedback suppression of the MAPK signaling with KRAS inhibition. Most clinically relevant, however, is the downstream activation of *KRASG12C* from high levels of EGFR signaling. Therefore, it was postulated, and shown in KRAS CRC cell line analysis, that concomitant EGFR and *KRAS G12C* blockade overcomes secondary resistance to anti-EGFR antibodies, increasing cell death rate (101). This concept led to the KRYSTAL-1 trial, a phase 1-2 open label non-randomized trial of patients with pre-treated *KRAS G12C* mutated CRC in which patients were provided adagrasib, an oral small molecule inhibitor of *KRAS G12C* protein in combination with cetuximab or adagrasib monotherapy (**Table 1**). The combination therapy had a statistically significant higher response rate (46% *vs* 19%), median duration of response (7.6 *vs* 4.3 months), and median PFS (6.9 *vs* 5.6 months), with a lower percentage of grade 3 or 4 treatment related adverse events (102). Additionally, the currently ongoing phase II clinical trial CodeBreaK 101, subprotocol H is attempting to combine sotorasib with panitumumab (**Table 1**) (100). Targeted therapy of KRASG12C in combination with ant-EGFR therapy appears to be a promising late-line therapy in patients harboring this mutation, improving response rates and PFS in patients that otherwise would be very limited in remaining effective treatment options.

## DNA mismatch repair and microsatellite unstable tumors

The advent of immune checkpoint and its application in tumors deficient in mismatch repair (dMMR) has resulted in significant improvement not only in the treatment efficacy but quality of life of the estimated 15% of colorectal cancer patients with this alteration. Mismatch repair genes including MLH1 (human mutL homolog 1), MSH2 (human mutS homolog 2), MSH6 (humab mutS homolog 6) and PMS2 (human postmeiotic segregation 2) are committed to mending errors during DNA replication such as incorrect base pairing, deletions or insertions (103–105). Up to eighty percent of cases are sporadic in etiology, secondary to epigenetic influences *via* the lack of methylation or excess methylation of DNA or DNA promotor regions respectively (106–110). This is in contrast to germline mutations within MMR genes, seen in hereditary forms of dMMR, leading to lack gene expression as seen in Lynch syndrome (111, 112). MMR deficiency lends tumor cells to amass large amounts of errors within DNA, developing microsatellites of repeated nucleotide bases that can result in significant abnormalities in DNA promoters responsible for cell proliferation, hence the term high microsatellite instability or MSI-H (108, 113).

The use of immunotherapy, specifically, anti-programmed cell death 1 monoclonal antibodies (anti-PD-1) in mCRC was first demonstrated in the treatment refractory setting. Specifically, in the 2015 phase II study of pembrolizumab monotherapy at 10mg/kg every 2 weeks in patients with treatment refractory dMMR mCRC, dMMR metastatic noncolorectal and MMR proficient (pMMR) mCRC, those with dMMR mCRC demonstrated an 89% DCR, and 50% ORR, relative to pMMR patients who had 16% DCR and 0% ORR. At a nearly 6-month treatment duration, PFS and OS were not reached in the dMMR group *vs* a PFS and OS of 2.3 months and 7.6 months respectively in the pMMR group (114). Based on these results, the authors opened the phase II open label, multicenter KEYNOTE 164 trial (**Tables 1**, **2**). In this study, patients with treatment refractory dMMR mCRC were provided pembrolizumab at 200mg every 3 weeks. OR was 33% in patients with ≥2 lines of therapy (cohort A) or ≥ 1 line of therapy (cohort B), with median OS of 31.4 months (95% CI 21.4 to 8.1months) in cohort A and not reached (95% CI 19.2 to not reached) in cohort B at a median follow up of 31.3 months (115). These results significantly contributed to the FDA approval of pembrolizumab for patients with dMMR or MSI-H disease that progressed on prior cytotoxic chemotherapy.

**Table 2:** Treatment of Metastatic MSI-H or dMMR.

*Trial*	KEYNOTE 177	CheckMate 142
Phase	Randomized; III	Non-randomized; II
Eligibility	Untreated MSI-H or dMMR Metastatic disease	MSI-H or dMMR Untreated in the metastatic setting*
Line of Therapy	1st	1+ (1^st^ treatment in metastatic disease)
Intervention vs Control	Pembrolizumab 200mg q3weeks vs mFOLFOX6 or FOLFIRI q2weeks +/- cetuximab q1week or +/-bevacizumab q2weeks	Nivolumab 3 mg/kg q2weeks and Ipilimumab 1 mg/kg q6weeks
Enrollment	307 patients -Pembrolizumab: 153 -Chemotherapy: 154	45 patients
Crossover Allowed	Yes	N/A
Objective Response Rate	Pembrolizumab: 44% Chemotherapy: 33%	Investigator assessment: 69% Blinded central review: 62%
Progression Free Survival	Pembrolizumab: 16.5 months (95% CI 5.4-38.1) Chemotherapy: 8.2 months (95% CI 6.1-10.2)	Not reached 24 month PFS rate: 73.6%
Median Overall Survival*	Pembrolizumab: Not Reached(95% CI 49.2–NR) Chemotherapy: 36.7 months (95% CI 27.6–NR)	Not reached 24-month OS rate: 79.4%
Grade ≥ 3 AE	Pembrolizumab: 22% No treatment related deaths Chemotherapy: 66% Treatment related deaths: 1	22% No treatment related deaths

-KEYNOTE 177: Median follow up of 44.5 months.

-CheckMate 142: Median follow up 29 months.

*40% of patients had prior adjuvant or neoadjuvant therapies. N/A means not applicable.

Similarly, the PD-1 inhibitor, nivolumab, received expedited approval the same year for treatment refractory dMMR or MSI-H mCRC based on the CheckMate 142 trial, in addition to its combination with ipilimumab (cytotoxic T-lymphocyte associated antigen-4 inhibitor) the following year (**Tables 1**, **2**). In this phase II, non-randomized multicohort study, patients with progressive dMMR mCRC were provided 3mg/kg nivolumab every 3 weeks and ipilimumab (CTLA-4 inhibitor) 1mg/kg every 3 weeks for 4 doses followed by nivolumab 3mg/kg every 2 weeks until disease progression, death or unacceptable toxicity, or nivolumab monotherapy 3mg/kg every 2 weeks. First analyzed and reported were the results from the nivolumab monotherapy arm, indicating that at a median follow up of 12 months, 69% (95% CI 57-79) of the 74 patients had disease control for 12 weeks or longer and 31.1% (CI 20.8-42.9) had objective response (116). In the cohorts that received both nivolumab and ipilimumab, a 4 year follow up has been reported. At a median follow up of 50.9 months, OR was seen in 65% of patients (95% CI 55%-73%), and a disease control of greater than or equal to 12 weeks was seen in 81% of patients (95% CI 72%-87%). Although median PFS and OS were not reached, 48-month PFS and OS percentage were 53% (95% CI 43-62) and 71% (95% CO 61-78) respectively (117). Notably, responses mentioned in both CheckMate 142 analyses responses were seen regardless of PD-L1 status, *BRAF* or *KRAS* status. Although no direct comparison has been made between dual checkpoint inhibitors versus immunotherapy monotherapy, risks and benefits must be weighed in this treatment refractory setting given the higher frequency of immune related toxicity with combination therapy (118).

Importantly, however, it has been concluded that early identification of MSI-H/dMMR tumors and subsequent first line treatment with immunotherapy in mCRC has improved responses relative to first line cytotoxic chemotherapy. First, the use of pembrolizumab monotherapy was analyzed in the phase III open label, randomized trial, assigning untreated patients with dMMR/MSI-H mCRC to pembrolizumab 200mg every 3 weeks or standard of care chemotherapy with 5-FU based therapy with oxaliplatin or irinotecan. Of note, cross over to pembrolizumab was allowed after disease progression. At a median follow up of 32.4 months, OR was seen in 43.8% in the pembrolizumab cohort *vs* 33.1% in those treated with chemotherapy. PFS was significantly longer in the pembrolizumab cohort versus chemotherapy at 16.5 months *vs* 8.2 months respectively (HR 0.6, 95% CI 0.95 0.45 to 0.80). Those patients that had complete or partial response to therapy, 83% of patients in the pembrolizumab arm had continued response at 24 months relative to 35% of patients in the chemotherapy arm. Importantly, pembrolizumab resulted in less grade 3-5 adverse events relative to standard chemotherapy (22% *vs* 66%), and improved health related quality of life (119, 120). There was a trend toward overall survival benefit with the use of pembrolizumab, but this result was skewed due to 60% of patients treated with chemotherapy crossing over to pembrolizumab (121). Due to these results, the American Society of Clinical Oncology 2022 guidelines recommended that patients with dMMR mCRC should be offered pembrolizumab monotherapy as first line therapy if eligible (122).

A subset of CheckMate 142 analyzed 45 patients with MSI-H/dMMR mCRC that were treatment naive. These patients were treated with nivolumab 3mg/kg every 2 weeks plus ipilimumab 1mg/kg every 6 weeks, with both drugs continued until disease progression. At a median follow up of 29 months, disease control rate was 84% (95 CI 70.5 vs 93.5), and ORR was 69% (95% CI 53-82), with 13% of patients having a complete response. Median PFS and OS was not reached (123). With these results, nivolumab with or without ipilimumab are considered first line therapy options in patients with dMMR/MSI-H mCRC, however, pembrolizumab remains the preferred regimen.

Under active study is the use of immunotherapy for patients with metastatic, chemo-refractory, microsatellite stable (MSS) disease. Early phase studies suggest that combination of the multikinase inhibitor regorafenib with immunotherapy provide objective response and improvement in PFS and OS. **Table 3** compares completed phase I and II studies of this combination along with a phase Ia/Ib study of the novel therapy botensilimab, an antibody directed against T-cell receptor cytotoxic T-lymphocyte-associated antigen 4 in combination with the novel monoclonal PD-1 antibody balstilimab (124–127).

**Table 3:** Early Phase and Developing Studies of Immunotherapy in MSI-Stable Disease.

*Trial*	NCT 04126733	NCT 04362839	NCT 03860272**
Phase	Open Label; II	Non-randomized; I	Expanded phase Ia/Ib
Eligibility	Previously treated MSS/pMMR Metastatic disease	Previously treated MSS/pMMR Metastatic disease	Previously treated MSS/pMMR Metastatic disease
Line of Therapy	>2 for RAS mutant >3 RAS wild type	1+	1+
Intervention vs Control	Regorafenib 80 mg/day 3 weeks on, 1 week off *increase to 120mg daily on C2 if well tolerated and Nivolumab 480mg q4 weeks	Regorafenib 80mg/day (Recommended phase II dosing determination) 3 weeks on, 1 week off and Nivolumab 240mg q2weeks and Ipilimumab 1 mg/kg q6weeks	Botensilimab 1 mg or 2 mg (or 150mg) q6 weeks and Balstilimab 3mg/kg (or 450mg) q2 weeks
Enrollment	94 patients 70 treated	39 patients	59 patients
Crossover Allowed	N/A	N/A	From monotherapy to combination
Objective Response Rate	7% (p = 0.27)	27.6% (all patients) 36.4% (without liver metastasis)	22% (all patients) (95% CI 12-35) -1 mg/kg: 38% -2 mg/kg: 20%
Progression Free Survival	1.8 months (95% CI 1.8-2.4)	4 months (all patients) (IQR 2-9 months) 5 months (without liver metastasis) (IQR 2-11 months)	Not available
Median Overall Survival	11.9 months (95% CI 7.0-not evaluable)	20 months (IQR 7 months – not estimable) >22 months	12 month OS: 61% (95% CI 42-75)
Grade ≥ 3 AE	Grade 3: 40% Grade 4: 3% Grade 5: 3%	N/A *No dose de-escalation needed at 80mg	Grade 3: 32% Grade 4: 2% Grade 5: 0%

IQR, Interquartile range.

**Open label Phase II multicenter study is currently active and enrolling. N/A means not applicable.

## Discussion

The utilization of molecular and genetic tumor analysis of patients with mCRC has become increasingly paramount to optimize first line treatment, allow for thoughtful pursuit of subsequent line therapy, and improve overall survival for patients with mCRC. It has become evident that proper use of adjunctive therapies added to established cytotoxic chemotherapy, particularly monoclonal antibodies, can provide meaningful impact on the survival to patients with mCRC. Continued investigation of novel mutational targets is necessary to further the quality of life and survival benefits already demonstrated by harnessing the inhibition of HER2, KRAS G12C, BRAF, VEGF and EGFR. As additional therapeutic molecular and genetic targets are discovered, easily accessible and rapidly resulting testing modalities, such as next generation sequencing, need to be made available for all oncology centers to provide optimal and equitable oncology care to all patients.

## Author contributions

All authors contributed to the article and approved the submitted version.
